# Mutation Profile of SARS-CoV-2 Genome Sequences Originating from Eight Israeli Patient Isolates

**DOI:** 10.1128/MRA.01387-20

**Published:** 2021-01-07

**Authors:** Galia Zaide, Inbar Cohen-Gihon, Ofir Israeli, Dana Stein, Ohad Shifman, Shay Weiss, Irit Simon, Orly Laskar, Adi Beth-Din, Anat Zvi

**Affiliations:** a Department of Biochemistry and Molecular Genetics, Israel Institute for Biological Research, Ness-Ziona, Israel; b Department of Infectious Diseases, Israel Institute for Biological Research, Ness-Ziona, Israel; c Department of Biotechnology, Israel Institute for Biological Research, Ness-Ziona, Israel; Queens College

## Abstract

We report the genome sequences and the identification of genetic variations in eight clinical samples of severe acute respiratory syndrome coronavirus 2 (SARS-CoV-2). Samples were collected from nasopharyngeal swabs of symptomatic and asymptomatic individuals from five care homes for elderly and infirm persons in Israel. The sequences obtained are valuable, as they carry a newly reported nonsynonymous substitution located within the nucleoprotein open reading frame.

## ANNOUNCEMENT

Shortly after a severe acute respiratory syndrome emerged in Wuhan, China, in December 2019 (1, 2), a new *Betacoronavirus* strain of the *Coronaviridae* family named severe acute respiratory syndrome coronavirus 2 (SARS-CoV-2) was identified as the etiological agent of a disease that was later termed coronavirus disease 19 (COVID-19) (2, 3). In this report, we describe the sequencing of eight SARS-CoV-2 samples obtained from specimens from five care homes for elderly and infirm persons in Israel. This study is in line with the ethical statement of the associate director general of the Israeli Ministry of Health. The individuals were initially identified as positive for COVID-19 by reverse transcriptase quantitative PCR (RT-PCR) and exhibited low cycle threshold (*C_T_*) values ranging from 12.8 to 16.8, implying a high viral load. Partial clinical information indicated that at least 2 of the 8 samples (i.e., EPI_ISL_594157 and EPI_ISL_594158) originated from asymptomatic individuals.

Samples were collected directly from swabs, and RNA was extracted with a QIAamp viral RNA minikit (Qiagen) according to the manufacturer’s protocol, using 60 µl of AVE buffer for elution. A SMARTer stranded total RNA-Seq pico input mammalian v2 kit (TaKaRa) was used for library construction prior to sequencing on a MiSeq instrument (Illumina). Whole-genome, paired-end sequencing was conducted in a duplex or triplex format with a read length of 150 nucleotides.

FastQC (https://www.bioinformatics.babraham.ac.uk/projects/fastqc) with default settings was used for quality control of the data. Trimming and removal of low-quality reads were performed using Trim Galore! v0.6.3 (http://www.bioinformatics.babraham.ac.uk/projects/trim_galore/) with default settings. Bowtie 2 (4) with default parameters was used for filtering of the results and for mapping the filtered reads against the reference Wuhan strain (GenBank accession number NC_045512). Reads mapped to SARS-CoV-2 were used as input data for the SPAdes assembler v3.13.0 (5) or the DNAStar software (SeqMan NGen v17.0; DNAStar, Madison, WI), resulting in a single contig for each sample. The genomic features of the samples are summarized in Table 1. Variant calling was performed using the SAMtools software package (6) with default parameters; a variant quality score cutoff of 100 was applied for all samples. A phylogenetic analysis generated using Nextstrain (7), rooted relative to the early samples from Wuhan, revealed that two of the eight samples (i.e., EPI_ISL_594155 and EPI_ISL_594156) belong to clade 20B, while the rest belong to clade 20C.

**TABLE 1:** Genome features of eight SARS-CoV-2 clinical samples

Sample	Genbank accession no.	Total no. of reads	No. of mapped reads	Avg coverage (×)	Assembly length (bp)	Overall G+C content (%)
NH-MA	MW227568	1,151,461	6,406	37	29,894	37.94
NH-GD3	MW201578	2,299,402	20,459	128	29,895	37.94
NH-NM	MW193889	1,759,712	51,125	214	29,899	37.94
NH-GD2	MW201577	1,245,093	28,057	143	29,870	37.94
NH-GD1	MW237708	1,099,546	14,081	62	29,895	37.94
NH-AS	MW201576	3,106,246	36,205	245	29,927	37.92
NH-M2	MW194121	6,485,364	59,944	297	29,930	37.91
NH-M1	MW228070	3,982,658	16,558	113	29,942	37.99

**FIG 1:**
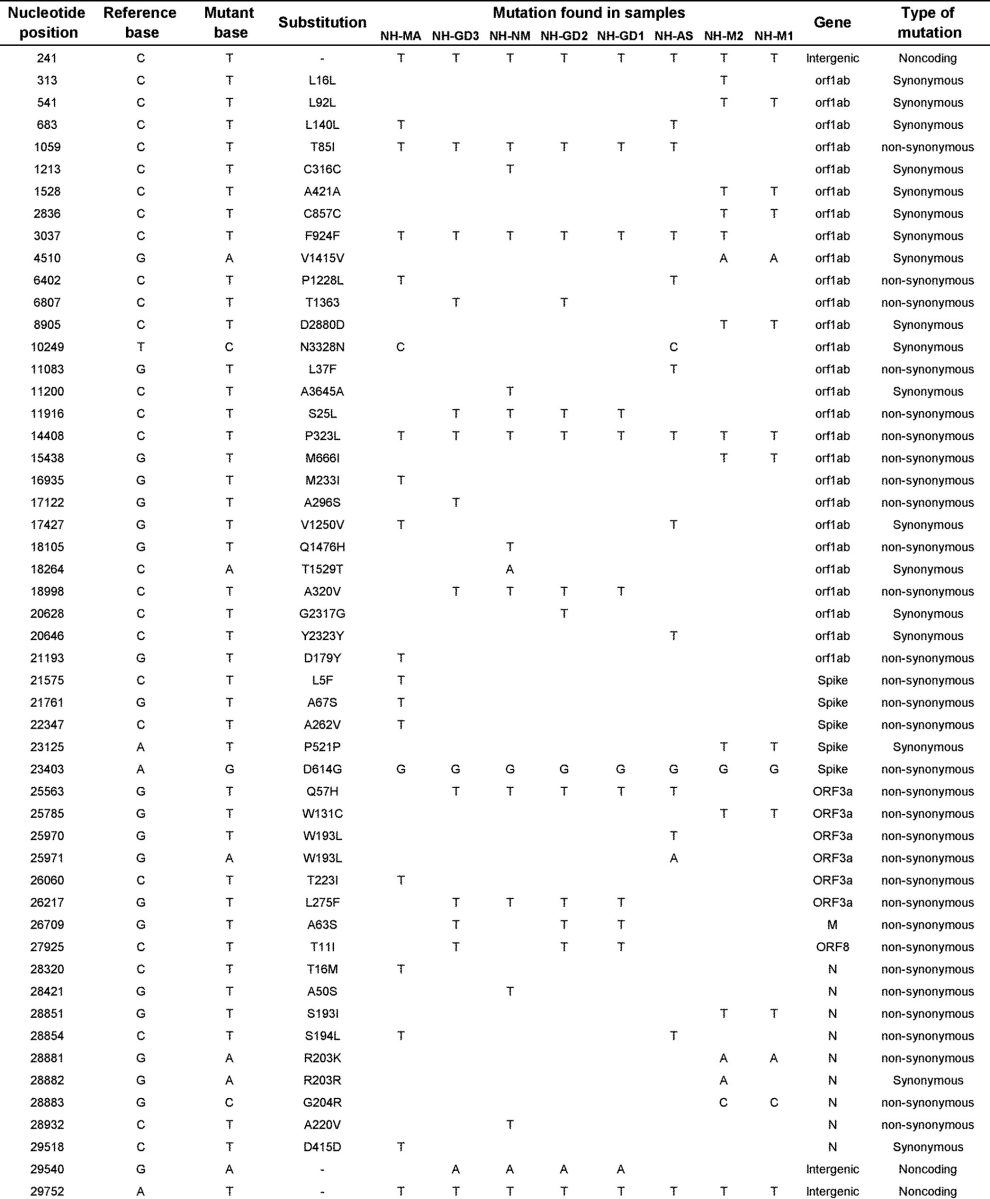
Mutations detected in the SARS-CoV-2 sequences generated in this study. The reference base was retrieved from the reference Wuhan strain (GenBank accession number NC_045512). Samples are named according to the isolate feature in the corresponding GenBank record (see Table 1).

The variant calling process revealed a total of 52 unique single-nucleotide polymorphism (SNP) replacements. A total of 31 substitutions were nonsynonymous, 4 of which mapped to the Spike coding region; 18 substitutions were of the synonymous type, and the remaining 3 substitutions occurred in noncoding regions (Fig. 1). The eight samples share one common mutation in an intergenic region (position 241, C to T) and two common mutations in coding regions (positions 23403, A to G, and 14408, C to T), resulting in the well-documented D614G substitution and the P323L replacement, respectively (Fig. 1). Apart from the abundant D614G replacement, six other nonsynonymous abundant replacements found in this study (i.e., T85I, L37F, S25L, P323L, A320V, and Q57H; Fig. 1) were previously reported as a result of hot spot mutations (8–10).

While most of the nonsynonymous replacements were previously reported (11), the A50S substitution (located in the nucleocapsid protein) identified in the EPI_ISL_594161 sample, was not documented before (GISAID [12, 13], as of November 2020).

Although several papers documented a list of viral factors that are correlated with COVID-19 severity (9, 14–16), there is still more to it than meets the eye. Thus, mapping and identification of new mutations may contribute to a better understanding of the viral factors related to clinical outcomes of the disease.

### Data availability.

The genome sequences have been deposited at the GISAID EpiCoV coronavirus SARS-CoV-2 platform database under the identifiers EPI_ISL_594155, EPI_ISL_594156, EPI_ISL_594157, EPI_ISL_594158, EPI_ISL_594159, EPI_ISL_594160, EPI_ISL_594161, and EPI_ISL_594162 and in the NCBI GenBank database under the accession numbers MW228070, MW194121, MW201576, MW227568, MW237708, MW201577, MW193889, and MW201578. The raw reads have been submitted to the NCBI Sequence Read Archive under the study reference number PRJNA672811.
